# Complete Genome Sequence of Morganella morganii CTX51T, Isolated from a Human Cecal Adenocarcinoma

**DOI:** 10.1128/mra.00066-22

**Published:** 2022-03-07

**Authors:** Marija Stepanovica, Martha A. Zepeda-Rivera, Adam S. McGlinchey, Alexander A. Baryiames, Dakota S. Jones, Kaitlyn D. LaCourse, Susan Bullman, Christopher D. Johnston

**Affiliations:** a Vaccine and Infectious Disease Division, Fred Hutchinson Cancer Research Center, Seattle, Washington, USA; b Human Biology Division, Fred Hutchinson Cancer Research Center, Seattle, Washington, USA; University of Maryland School of Medicine

## Abstract

We report the complete genome sequence of Morganella morganii CTX51T, a strain isolated from the resected tumor of a patient with cecal colorectal adenocarcinoma of the cecum. The genome comprises a circular chromosome of 4.19 Mbp, with an overall GC content of 50.4% and one circular plasmid of 8.48 kbp.

## ANNOUNCEMENT

Morganella morganii, a Gram-negative, facultative anaerobe, is a commensal of the human intestinal tract and an opportunistic pathogen (1) in a wide range of nosocomial and community-acquired infections (2–6). Here, we report the isolation of M. morganii CTX51T, a strain cultivated from a colorectal cancer tumor.

CTX51T was isolated from a surgically resected tumor from a treatment-naive, male colorectal cancer patient diagnosed with adenocarcinoma in the cecum. Briefly, tissue sections were minced with a scalpel, spread onto fastidious anaerobe agar plates (Oxoid, Thermo Fisher Scientific, USA) supplemented with 10% defibrinated horse blood (Lampire Biological Laboratories, Fisher Scientific, USA) (FAA + 10% DHB), and incubated at 37°C for 48 h under anaerobic conditions (AnaeroGen Gas Generating Systems, Oxoid, Thermo Fisher Scientific). The resulting bacterial colonies were picked and streak purified. CTX51T was cultured under the above conditions, and high-molecular-weight genomic DNA was extracted from plate-grown colonies using the MasterPure DNA purification kit (Epicentre, Lucigen, USA). Single-molecule real-time sequencing (SMRT-Seq) (PMID: 19023044) was carried out on a Sequel I instrument (Pacific Biosciences, USA). Qubit double-stranded DNA (dsDNA) broad range (BR) assays (Thermo Fisher Scientific) were used to determine the DNA concentration, and 3 μg genomic DNA was sheared to an average size of 12 kb using a g-TUBE device (Covaris, USA). Libraries were generated using the SMRTbell Express template prep kit 2.0 (Pacific Biosciences), and the pooled libraries were size selected using the BluePippin system (Sage Sciences, USA) at a 4-kb minimum threshold. The Pacific Biosciences SMRT Analysis pipeline version 9.0.0.92188 was first used to process the sequencing reads; then, the reads were assembled using Microbial Assembler with default parameters, which includes an error correction step for chromosomal contiguity and rotation to place the first nucleotide at the chromosomal replication gene, *dnaA*. The sequencing reads were processed using the SMRT Analysis pipeline version 9.0.0.92188, yielding 4,634 reads for assembly with an *N*_50_ value of 11,806 bp, a mean read length of 11,203 bp, and a coverage of ∼110×. Genome assembly resulted in a chromosomal contig of 4,185,431 bp and a putative plasmid contig of 8,480 bp, with average GC contents of 50.4% and 40.5%, respectively.

**FIG 1:**
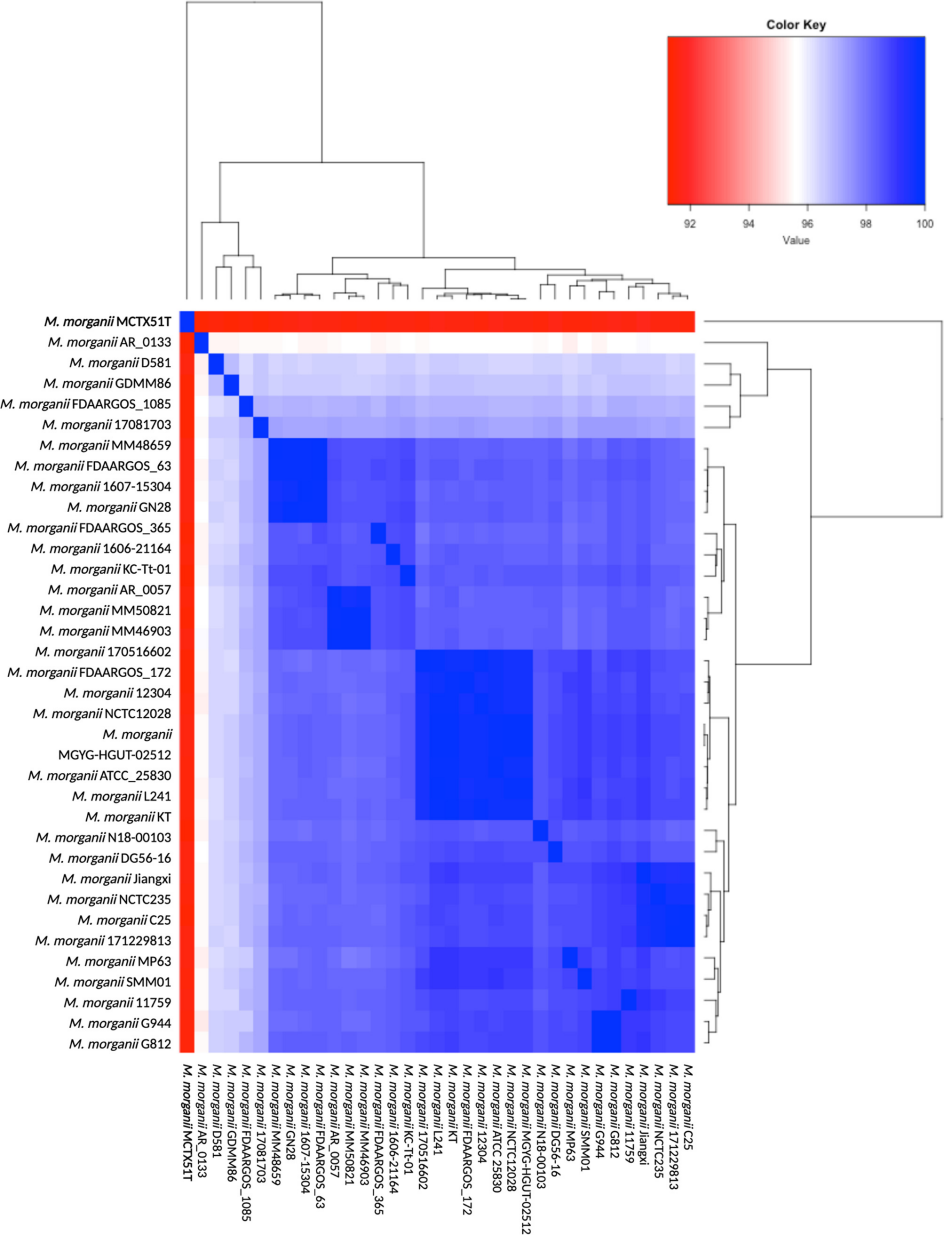
Heatmap of average nucleotide identity (ANI) values. The ANI of M. morganii CTX51T was compared to 34 publicly available M. morganii genomes (Table 1) using JSpeciesWS (10). Blue indicates higher ANI values, whereas red indicates lower ANI values. The heatmap was made using the heatmap.2 function from the gplots package in RStudio version 1.4.1103 (11), and the final figure was created at https://biorender.com/.

Classification of CTX51T as Morganella morganii is based on 16S rRNA gene sequencing (7) and average nucleotide identity (ANI) analysis (PMID: 17220447 and PMID: 19855009) (Fig. 1; Table 1). Genome annotation using the NCBI Prokaryotic Genome Annotation Pipeline (PGAP) (8) identified 3,863 coding sequences and 108 RNAs. Methylome annotation via the Restriction Enzyme Database (REBASE) (9) identified one putative type I restriction-modification (RM) system, four type II RM systems, and two orphan methyltransferase systems. Two modified motifs, G^m6^ATC and GC^m6^ANNNNNNRTGT, were detected as being methylated at 96.1% and 90.2%, respectively. Additional analysis using CRISPRDetect (12) and CRISPRCasTyper (13) found no CRISPR arrays or Cas genes within the CTX51T genome.

**TABLE 1:** Publicly available M. morganii genome assemblies used for ANI analysis^*a*^

Strain	GenBank accession no.	Isolation source^*b*^
11759	GCA_018141465.1	Urine
12304	GCA_018802585.1	NA
81703	GCA_018802525.1	NA
516602	GCA_018802405.1	NA
229813	GCA_011465095.1	Blood
621164	GCA_018802565.1	NA
715394	GCA_018802545.1	NA
AR_0057	GCA_002968775.1	NA
AR_0133	GCA_003071325.1	NA
ATCC 25830	GCA_006094455.1	NA
ZJC25	GCA_018802505.1	NA
ZJD581	GCA_018802485.1	NA
DG56-16	GCA_003573445.1	Liver
FDAARGOS_1085	GCA_016727445.1	NA
FDAARGOS_172	GCA_001558895.2	Urine
FDAARGOS_365	GCA_002386305.1	Stool
FDAARGOS_63	GCA_000783955.2	Wound
ZJG812	GCA_018802465.1	NA
ZJG944	GCA_018802445.1	NA
GDMM86	GCA_016618235.1	Environment
GN28	GCA_018802425.1	NA
Jiangxi	GCA_013378135.1	NA
KC-Tt-01	GCA_002891475.1	Pericardial fluid
KT	GCA_000286435.2	Blood
L241	GCA_003955965.1	Feces
MGYG-HGUT-02512	GCA_902387845.1	Gut
MM46903	GCA_016939515.1	Pressure ulcer
MM48659	GCA_016939575.1	Urine
MM50821	GCA_016939635.1	Sputum
MP63	GCA_010748915.1	Wastewater
N18-00103	GCA_010365245.1	Sputum
NCTC12028	GCA_900478755.1	Stool
NCTC235	GCA_900635025.1	NA
SMM01	GCA_015698325.1	Urine

a These assemblies were used for ANI and 16S rRNA analysis with M. morganii CTX51T.

b NA, not available.

Several putative virulence factors were identified, including Tc toxins, fimbrial adhesins, and both type III and type VI secretion system components (14, 15). Elevated levels of M. morganii antibiotic resistance have been reported (1, 6, 16–19); as such, we analyzed CTX51T using the Comprehensive Antibiotic Resistance Database (CARD) (20), which identified 282 chromosomal genes associated with resistance to fluoroquinolones, beta-lactams, macrolides, and tetracyclines.

Currently, 34 M. morganii genome assemblies are publicly available. CTX51T is the first complete M. morganii genome sequence from a cancer-associated niche and may therefore help advance our clinical understanding of this species.

### Data availability.

The BioProject accession number for this genome, as well as that for many other human-associated bacterial isolates, is PRJNA549513. The RefSeq assembly accession number is GCF_020911745.1. The genome sequence was deposited in GenBank under the accession number CP076623. The base modification files are available with the GenBank accession and methylome analysis at REBASE under organism number 49937. The SRA accession number for the raw read data is SRR17841631.
